# Pulmonary Arterial Hypertension in a Patient With a Lung Mass: Any Link?

**DOI:** 10.7759/cureus.75729

**Published:** 2024-12-15

**Authors:** Abdullah M Alharbi, Mohammed S Alqarni, Abdulkareem A Alkahtani, Bader Alghamdi

**Affiliations:** 1 Pulmonology, National Guard Hospital, King Abdulaziz Medical City, Jeddah, SAU; 2 Internal Medicine, King Abdullah International Medical Research Center, Jeddah, SAU; 3 Internal Medicine, National Guard Hospital, King Abdulaziz Medical City, Jeddah, SAU; 4 Medical Imaging, King Abdulaziz Medical City, Jeddah, SAU; 5 College of Medicine, King Saud Bin Abdulaziz University for Health Sciences, Jeddah, SAU; 6 Pulmonology, King Abdulaziz Medical City, Jeddah, SAU

**Keywords:** chronic thromboembolic pulmonary hypertension (cteph), cteph, egfr mutation, granulomatous disease, osimertinib, pulmonary embolism, pulmonary hypertension

## Abstract

A 52-year-old female patient with a history of atrial septal defect repair presented with progressive dyspnea and echocardiographic findings suggestive of pulmonary hypertension (PH). Incidentally, a lung mass was discovered on computed tomography (CT). Initial evaluation revealed World Health Organization functional class III symptoms and significant weight loss. Diagnostic workup included echocardiography, pulmonary function tests, CT chest, computed tomography pulmonary angiography, right heart catheterization, and positron emission tomography. Supraclavicular lymph node biopsy confirmed adenocarcinoma of pulmonary origin with positive epidermal growth factor receptor (EGFR) mutation. Right heart catheterization demonstrated vasoreactive pre-capillary PAH. The patient was initiated on nifedipine, which was gradually titrated. Subsequent ventilation-perfusion scan revealed chronic thromboembolic pulmonary hypertension (CTEPH), leading to anticoagulation therapy. We are reporting this case of PAH which presented with features of Group 4 PH and had a sarcoid-like reaction.

## Introduction

Pulmonary hypertension (PH) encompasses a spectrum of conditions that contribute to elevated pressures within the pulmonary arteries, either through direct or indirect mechanisms [1]. Hemodynamically, PH is defined as a mean pulmonary arterial pressure (mPAP) of more than 20 mmHg. PH is divided into five principal groups based on its pathophysiological attributes, clinical characteristics, and therapeutic options [1]. PH represents a major public health concern worldwide. The global prevalence of PH exceeds 1% of the total population [2]. PH is a progressive disease that needs close monitoring of the patient and continuous risk stratification to intervene at appropriate time [2].

Regardless of the specific group, pulmonary arterial hypertension (PAH) is consistently linked to detrimental changes in the structure and function of the pulmonary blood vessels [3]. This adverse vascular remodeling process leads to obstruction, increased stiffness, and constriction of the pulmonary vasculature, which ultimately contributes to the elevated pressures observed in the pulmonary arteries across all forms of the disease [4-5].

Having concomitant lung cancer can exacerbate PH through both macrovascular (e.g., tumor macroembolism) and microvascular involvement (e.g., pulmonary tumor microembolism and pulmonary tumor thrombotic microangiopathy). The presence of dual conditions increases the patient's burden of illness and makes it difficult for the treating physician to decide what is best for the patient in terms of monitoring and treatment options [5].

We are reporting this case of PAH which presented with features of Group 4 PH and had a sarcoid-like reaction that has not been reported in the literature previously. Our objective is to present this rare case by applying therapeutic principles from our field and to propose a recommended approach for managing similar cases in the future.

## Case presentation

A 52-year-old female was referred to the PH clinic with progressive shortness of breath and echocardiography findings suggestive of PH; at the same time, she found to have incidental lung mass on the chest CT scan. Her medical history revealed that she underwent atrial septal defect repair 25 years ago. On initial assessment, she had progressive shortness of breath for one year, World Health Organization Functional Class (WHO-FC) III. There were no other cardiopulmonary symptoms. She reported significant weight loss of around 3 kg over three months. Other systematic reviews were unremarkable in terms of associated causes or exposure for PH. Besides these findings, she did not report any other medical issues. On physical examination, the patient was hemodynamically stable and afebrile. Regarding her vital signs her pulse was 78/min, blood pressure was 121/62 mmHg, temperature was 36.9 °C, and in-room air saturation of O_^2^_ was 96%. The chest was clear, and the abdomen was soft and lax without tenderness. There was no limb edema or signs of deep venous thrombosis. The rest of the physical examination was unremarkable. 

Laboratory workups for secondary causes of PH were all negative (Table 1). 

**Table 1:** Laboratory workups for secondary causes of pulmonary hypertension BNP: brain natriuretic peptide; ANA: antinuclear antibody; AMA: antimitochondrial antibody; RF: rheumatoid factor; HIV Ab/Ag: HIV antigen/antibody

Test	Result	Reference Range
BNP	60	Normal < 100
Schistosoma antibody	<1:16	Negative (titer < 1:16)
ANA	0.21	Negative (< 1:1)
AMA	<1:20	Negative (< 1:20)
Anti-smooth muscle antibody	<1:39	Negative (< 1:40)
RF	<10.90	Negative (< 15)
HIV Ab/Ag	Non-reactive	Non-reactive

**Figure 1:**
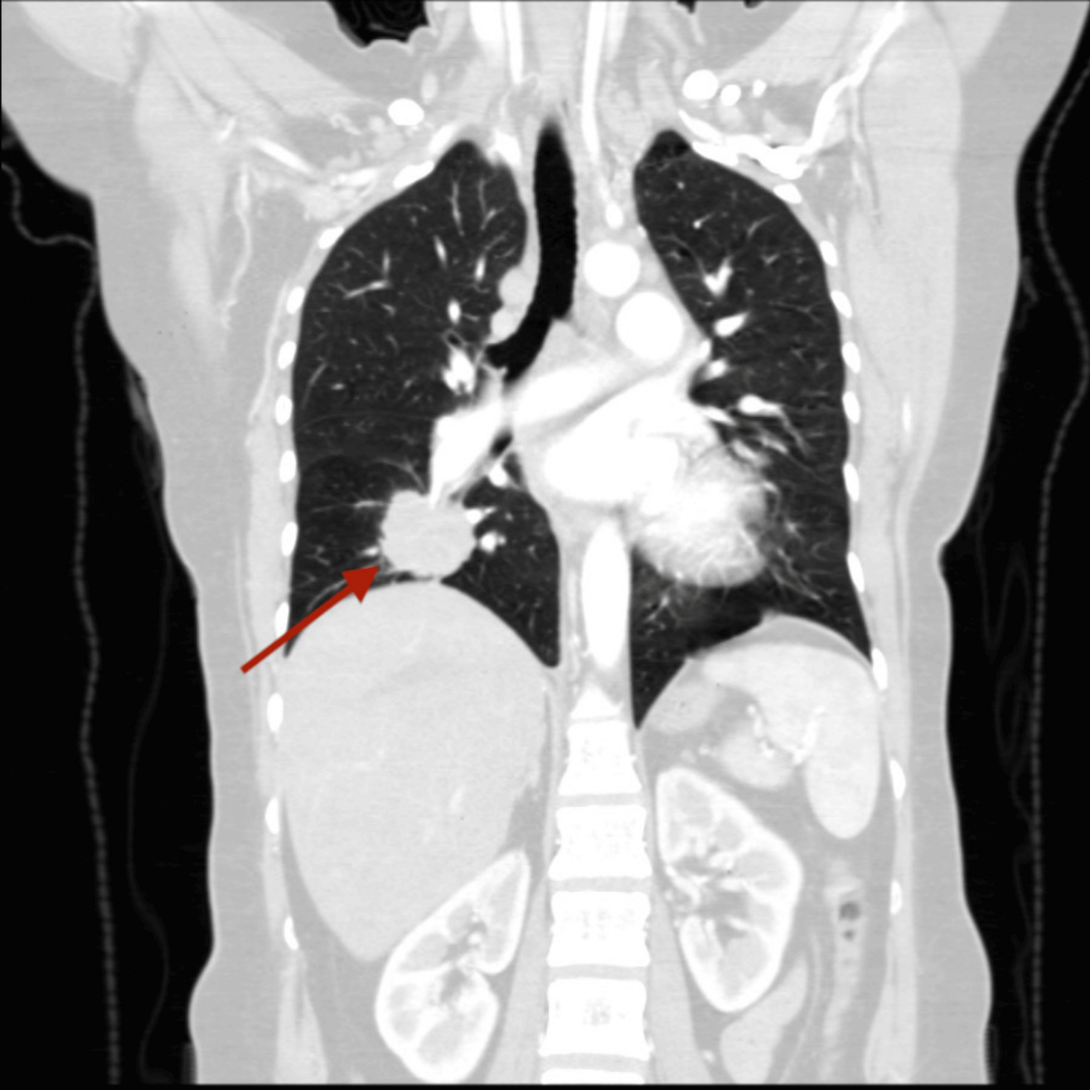
CT chest showed right lower lobe suspicious mass with bronchial obstruction/endobronchial extension, mediastinal lymphadenopathy

**Figure 2:**
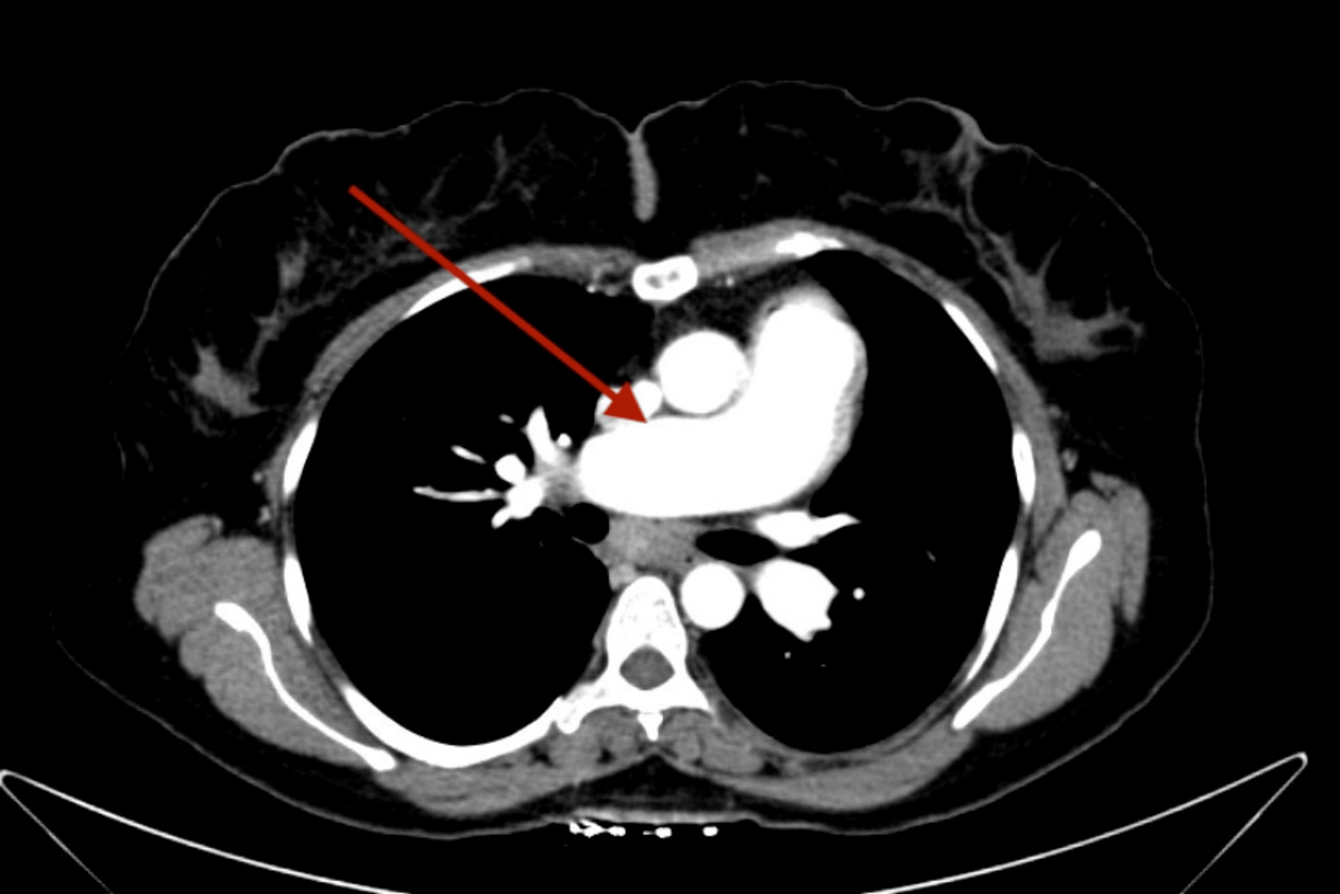
CT chest showed dilated pulmonary trunk

Doppler echocardiogram findings were normal left ventricle (LV), normal biatrial size, mildly dilated right ventricle (RV), tricuspid annular plane systolic excursion (TAPSE) of 15 mm, tricuspid regurgitation velocity (TRV) of 2.9 m/sec, and right ventricular systolic pressure (RVSP) of 37. The bubble study was negative. Pulmonary function test (PFT) showed mild restrictive patterns with normal DLCO (FEV1 53%, FVC 55%, FEV1/FVC Ratio 77%, TLC 77%, DLCO 93%). During the 6 Minute Walk Test (6MWT) she was able to walk 335 m without significant desaturation. CT chest showed a suspicious right lower lobe mass with bronchial obstruction/endobronchial extension, mediastinal lymphadenopathy, and dilated pulmonary trunk (Figures 1-2). 

Computed tomography pulmonary angiography (CTPA) did not show any signs of acute or chronic (PE). Ventilation perfusion (V/Q) was not done as the technetium was not available during the COVID period. Right heart catheterization (RHC) showed vasoreactive pre-capillary pulmonary hypertension (Table 2).

**Table 2:** Right heart catheterization mPAP: mean pulmonary arterial pressure; PCWP: pulmonary capillary wedge pressure; PVR: pulmonary vascular resistance; CO: cardiac index; SvO2: mixed venous oxygen saturation; WU: Wood units

Site	Pre-iloprost	Post-iloprost
mPAP	52 mmHg	37 mmHg
PCWP	11 mmHg	13 mmHg
PVR	9.6 WU	5.4 WU
CO	3.9 L/min	No change
SVo2	68%	

**Figure 3:**
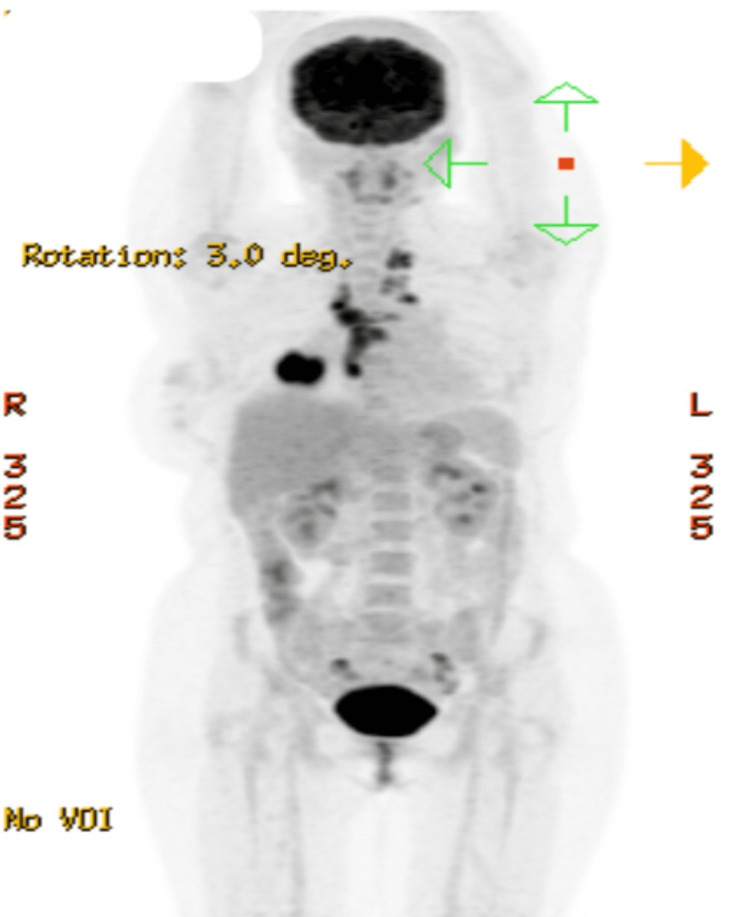
Positron emission tomography (PET) scan showed right lung lower lobe malignancy right hilar, mediastinal, and bilateral supraclavicular lymph node metastases

A positron emission tomography (PET) scan showed multiple mediastinal lymph nodes with supraclavicular involvement (Figure 3).

**Figure 4:**
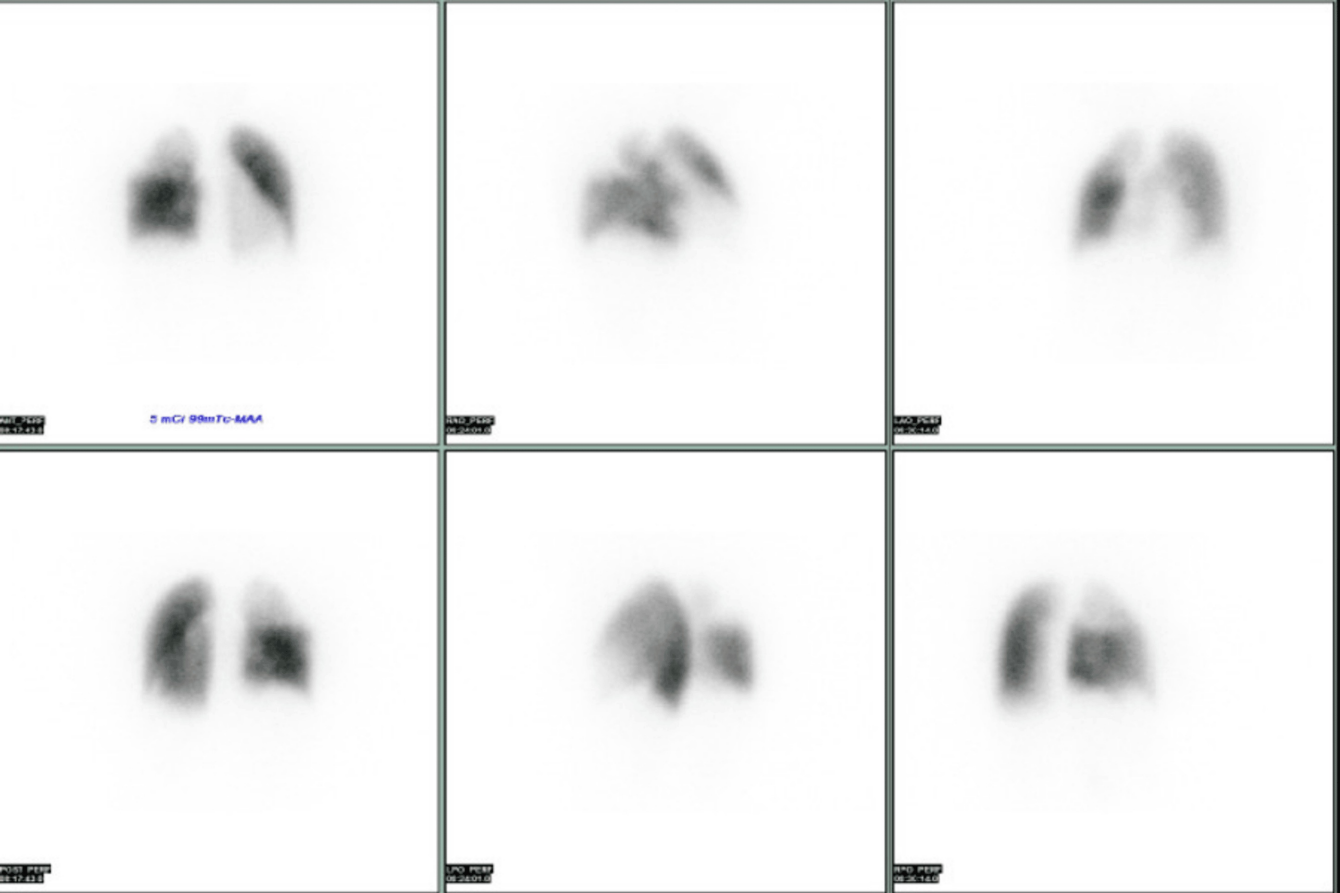
Ventiliation perfusion (V/Q) showed absent perfusion of posterior segment of right upper lobe and middle lobe and severe hypo-perfusion of apical segment of right upper lobe

She underwent a supraclavicular lymph node biopsy that showed adenocarcinoma of pulmonary origin, epidermal growth factor receptor (EGFR) positive. She was diagnosed with a case of vasoreactive pre-capillary PAH and Stage IIIB lung adenocarcinoma. Regarding the management of PH, she started on nifedipine and titrated up gradually. On follow up V/Q was done and showed a perfusion defect of the posterior segment of the right upper lobe and the middle lobe which confirmed chronic thromboembolic pulmonary hypertension (CTEPH) (Figure 4).

She was started on novel oral anticoagulants (NOACs). Her repeat RHC showed an improvement in hemodynamics as shown in Table 3. Currently, she is on nifedipine extended-release 150 mg OD, and she is in a low-risk group, WHO-FC I, BNP 40, and 6MWT 335 m.

**Table 3:** Post-treatment right heart catheterization mPAP: mean pulmonary arterial pressure; PCWP: pulmonary capillary wedge pressure; PVR: pulmonary vascular resistance; CO: cardiac index; SvO2: mixed venous oxygen saturation; WU: Wood units

Site	Pressure
mPAP	26 mmHg
PCWP	9 mmHg
PVR	2.2 WU
CO	5.02 L/min
SVo2	68%

Regarding adenocarcinoma, the patient was initiated on EGFR tyrosine kinase inhibitors, which resulted in a significant regression of the lung mass. The treatment with osimertinib was maintained at a dosage of 80 mg. The patient experienced disease progression-free survival for a duration of four years, at which point a new lymph node was identified during routine CT surveillance. A biopsy of this lymph node revealed non-ceasing granulomatous disease.

Following this finding, a multidisciplinary team convened to evaluate the situation; however, the decision was made not to commence steroid treatment due to the absence of clinical symptoms indicative of sarcoidosis. Instead, the granulomatous disease was attributed to the lung adenocarcinoma and/or potential side effects of the medication.

## Discussion

Acute vasoreactivity testing (AVT) is crucial for identifying acute vaso-responders and assessing the effectiveness of calcium channel blocker (CCB) therapy in patients with PAH who could potentially benefit from treatment [1]. Conversely, those who do not respond to AVT are unlikely to benefit from CCB therapy and if initiated; could face severe, potentially life-threatening side effects as a result [6,7]. The role of AVT in PH and the use of CCBs in PAH are critical considerations in the management of these conditions. While AVT and CCBs are not generally recommended for patients with CTEP, exceptions exist for those with idiopathic, drug-induced, or heritable forms of PAH. This recommendation is primarily based on limited evidence, suggesting that the efficacy of these treatments in CTEPH remains uncertain. However, it is essential to recognize that AVT and CCB therapy have shown significant clinical benefits and improved survival outcomes in specific patient populations with PAH. In particular, some studies indicate that a subset of CTEPH patients may experience symptomatic relief and hemodynamic improvements when treated with CCBs [1,8]. In our case report, the patient was initiated on CCB therapy after a positive response during AVT and demonstrated marked improvement in her clinical manifestations, despite exhibiting characteristics of CTEPH. Similarly, a cohort study conducted in 80 Chinese patients revealed that individuals with CTEPH who exhibited a positive response to AVT experienced superior survival outcomes compared to those who had a negative response during AVT [8].

In this case, the patient had lymph node enlargement, which was biopsied, revealing histopathological findings consistent with sarcoidosis; however, the patient did not present with any clinical symptoms typically associated with sarcoidosis. Additionally, the patient was found to have an EGFR mutation that responded positively to treatment with osimertinib. Kachalia et al. documented a case involving lung adenocarcinoma with an EGFR mutation coexisting with sarcoidosis. In that instance, the patient was initially diagnosed with sarcoidosis and showed a favorable response to corticosteroids. However, six months later, the lung adenocarcinoma had metastasized to the pleura, pericardium, and diaphragm, alongside the presence of an EGFR mutation [9]. Similarly, Zhao et al. reported a case of a patient with lung adenocarcinoma and a concurrent sarcoid-like reaction, where osimertinib was administered without the initiation of corticosteroid, as it was deemed unnecessary in that particular case [10].

## Conclusions

In this case, RHC revealed precapillary vasoreactive PAH), to which the patient showed an excellent response to CCB. However, it is critical to consider that her V/Q study indicated a perfusion defect in the context of active malignancy. This finding raises the possibility that Group 4 PH could have been a contributing factor to her condition. Differentiating the group or disease process implicated in her illness was of paramount importance.

Establishing whether PAH or Group 4 PH is involved is essential, as a targeted curative approach may be required. Further reevaluation and adjustment of her medication regimen should be considered based on the underlying disease process. This highlights the need for a comprehensive assessment to guide appropriate therapeutic strategies tailored to the underlying etiology of her PH.
